# Symptoms and quality of life in patients with suspected angina undergoing CT coronary angiography: a randomised controlled trial

**DOI:** 10.1136/heartjnl-2016-310129

**Published:** 2017-02-28

**Authors:** Michelle C Williams, Amanda Hunter, Anoop Shah, Valentina Assi, Stephanie Lewis, Kenneth Mangion, Colin Berry, Nicholas A Boon, Elizabeth Clark, Marcus Flather, John Forbes, Scott McLean, Giles Roditi, Edwin JR van Beek, Adam D Timmis, David E Newby

**Affiliations:** 1 British Heart Foundation Centre for Cardiovascular Science, University of Edinburgh, Edinburgh, UK; 2 Centre for Population Health Sciences, University of Edinburgh, Edinburgh, UK; 3 Institute for Cardiovascular and Medical Sciences, University of Glasgow, Glasgow, UK; 4 Norwich Medical School, University of East Anglia, UK; 5 University of Limerick, Limerick, Ireland; 6 National Health Service, Fife, UK; 7 William Harvey Research Institute, Queen Mary University of London, London, UK

## Abstract

**Background:**

In patients with suspected angina pectoris, CT coronary angiography (CTCA) clarifies the diagnosis, directs appropriate investigations and therapies, and reduces clinical events. The effect on patient symptoms is currently unknown.

**Methods:**

In a prospective open-label parallel group multicentre randomised controlled trial, 4146 patients with suspected angina due to coronary heart disease were randomised 1:1 to receive standard care or standard care plus CTCA. Symptoms and quality of life were assessed over 6 months using the Seattle Angina Questionnaire and Short Form 12.

**Results:**

Baseline scores indicated mild physical limitation (74±0.4), moderate angina stability (44±0.4), modest angina frequency (68±0.4), excellent treatment satisfaction (92±0.2) and moderate impairment of quality of life (55±0.3). Compared with standard care alone, CTCA was associated with less marked improvements in physical limitation (difference −1.74 (95% CIs, −3.34 to −0.14), p=0.0329), angina frequency (difference −1.55 (−2.85 to −0.25), p=0.0198) and quality of life (difference −3.48 (−4.95 to −2.01), p<0.0001) at 6 months. For patients undergoing CTCA, improvements in symptoms were greatest in those diagnosed with normal coronary arteries or who had their preventative therapy discontinued, and least in those with moderate non-obstructive disease or had a new prescription of preventative therapy (p<0.001 for all).

**Conclusions:**

While improving diagnosis, treatment and outcome, CTCA is associated with a small attenuation of the improvements in symptoms and quality of life due to the detection of moderate non-obstructive coronary artery disease.

**Trial registration number:**

NCT01149590.

## Introduction

We have reported the primary findings of the Scottish COmputed Tomography of the HEART (SCOT-HEART) trial^1^ and demonstrated that, when used in addition to standard care, CT coronary angiography (CTCA) clarified the diagnosis of angina pectoris due to coronary heart disease. This was associated with better selection of patients for invasive coronary angiography, more appropriate changes in therapy and a halving in the rates of fatal and non-fatal myocardial infarction.^2^

In the SCOT-HEART trial,^1^ one-third of patients were diagnosed with angina pectoris due to coronary heart disease. For the attending clinician, CTCA clarified the diagnosis of both coronary heart disease and angina pectoris due to coronary heart disease. However, CTCA had divergent effects on the frequency of these diagnoses with an increased rate in the diagnosis of coronary heart disease and an apparent reduction in the diagnosis of angina pectoris due to coronary heart disease. This was principally attributable to an increase in the diagnosis of non-obstructive coronary heart disease by CTCA.

There is substantial anxiety about the potential for, and consequences of, coronary heart disease in patients presenting with undifferentiated chest pain.^3^ Patients are looking for clear reassurance as well as a resolution to their symptoms. For the patients, the critical question is whether CTCA will help their symptoms and improve their future prognosis. Here, we assessed how CTCA affected the changes in patients' symptoms and quality of life at 6 weeks and 6 months after their attendance at the cardiology clinic.

## Methods

### Study design

The SCOT-HEART study was a prospective open-label parallel group multicentre randomised controlled trial that assessed the role of CTCA in patients with suspected angina due to coronary heart disease who attended a cardiology clinic. The study design has previously been described^4^ and the primary findings published.^1^ ^2^

### Participants

Participants were recruited from a dedicated cardiology chest pain clinic where they were referred with suspected angina due to coronary heart disease. A total of 4146 patients aged 18–75 were recruited as described previously.^1^ Participants were randomised 1:1 to standard care or standard care plus ≥64-slice CTCA using a web-based randomisation system. Standard of care included stress testing according to established local clinical protocols.

### Patient and public involvement

Patient and public involvement was incorporated throughout the trial development, conduct and completion. At study design stage, we assessed feasibility and received feedback on the potential acceptability of patient participation in the trial. During trial conduct, lay representatives contributed to the membership of the Trial Steering Committee. A lay member (EC) contributed to this article and is a coauthor.

### CT coronary angiography

CTCA images were assessed by at least two trained observers with excellent reproducibility.^5^ The overall results of the scan were defined as normal (<10% cross-sectional luminal stenosis), mild (10–50%) or moderate (50–70%) non-obstructive or obstructive (≥70% or >50% in the left main stem) coronary artery disease.

### Seattle Angina Questionnaire and Short Form 12

Angina symptoms were assessed with a self-administered UK version of the Seattle Angina Questionnaire.^6^ This questionnaire measures five clinically important domains of physical limitation, angina stability, angina frequency, treatment satisfaction and quality of life. Scores are expressed on a 0–100 scale with higher scores denoting better outcomes. It has been validated in patients with, or being assessed for, coronary heart disease and is responsive to therapeutic interventions.^6^ The questionnaire was performed at the baseline clinic attendance and then at 6 weeks and 6 months by post. For non-responders, telephone follow-up was performed where possible. To further assess and compare quality of life and health measures, participants were also asked to complete 12-item Short Form SF-12v2 Health Survey.^7^ This is a standardised instrument that measures eight health domains: physical functioning, role limitations due to physical health, bodily pain, general health, vitality (energy/fatigue), social functioning, role limitations due to emotional health and mental health (psychological distress and psychological well-being).

### Statistical analysis

Data are presented as mean±SE or mean differences with 95% CIs. Response rates to the Seattle Angina Questionnaires were compared between treatment arms with χ^2^ tests at each time point. Changes in the Seattle Angina Questionnaire and Short Form 12 were compared between treatment arms using t-tests. In patients allocated to CTCA, changes over time were compared according to the changes in the diagnosis of coronary heart disease and angina pectoris due to coronary heart disease using analysis of covariance. This was adjusted for the baseline score, centre and the minimisation variables (age, sex, body mass index, diabetes mellitus, atrial fibrillation, history of coronary heart disease and baseline diagnosis of angina pectoris due to coronary heart disease). We then performed univariable mixed models to investigate how symptom-related variables could help explain the changes in Seattle Angina Questionnaire scores at 6 months. All these analyses were adjusted for the baseline score. Finally, we extended the univariate model by building multivariable mixed models for each score using a stepwise approach. Statistical analysis was performed using SAS V.9.4. Statistical significance was defined as a two-sided p<0.05.

## Results

Of the 4146 patients recruited to the trial, 1432 (34.5%) patients were diagnosed with angina due to coronary heart disease after 6 weeks of follow-up. Seattle Angina Questionnaires were completed in 4142 (99.9%) at baseline, 3427 (82.7%) at 6 weeks and 3035 (73.2%) at 6 months. Although rates of completion were identical at baseline (99.9% for both), those assigned to CTCA were slightly more likely to complete the questionnaire than those allocated to standard care alone at both 6 weeks (84.7% vs 80.7%, p=0.0007) and 6 months (75.3% vs 71.1%, p=0.0018).

Baseline scores across the five domains of the Seattle Angina Questionnaire indicated mild physical limitation (74±0.4), moderate angina stability (44±0.4), modest angina frequency (68±0.4), excellent treatment satisfaction (92±0.2) and moderate impairment of quality of life (55±0.3). These domains were similar across the two trial groups at baseline (see online supplementary table 1).

10.1136/heartjnl-2016-310129.supp1 supplementary tables

### Changes in symptoms during follow-up

In general, symptoms improved across both study groups during follow-up (table 1) with the greatest improvements seen in angina frequency (p<0.001) and quality of life (p<0.001; see online supplementary table 1). There was little improvement in physical limitation or treatment satisfaction, perhaps reflecting the mild physical limitation and excellent treatment satisfaction seen at baseline.

**Table 1:** Changes from baseline in Seattle Angina Questionnaire after 6 weeks and 6 months in patients randomised to standard care plus CT coronary angiography (CTCA) and standard care alone

	All patients	Standard care+CTCA	Standard care	Difference (95% CIs)	p Value (for difference)
Change at 6 weeks	n=3427	n=1755	n=1672		
Physical limitation	−0.3±0.4 (2076)	−0.5±0.5 (1082)	−0.0±0.5 (994)	−0.72 (−2.08 to 0.63)	0.2957
Angina stability	16.3±0.6 (3190)	16.7±0.9 (1637)	15.8±0.9 (1553)	1.03 (−0.61 to 2.68)	0.2184
Angina frequency	11.5±0.4 (3264)	11.2±0.6 (1684)	11.8±0.6 (1580)	−0.84 (−2.20 to 0.54)	0.2277
Treatment satisfaction	−7.0±0.3 (3247)	−7.0±0.4 (1675)	−7.0±17.1 (1572)	0.03 (−1.07 to 1.14)	0.9525
Quality of life	9.3±0.4 (3261)	8.7±0.5 (1681)	9.9±0.6 (1580)	−1.31 (−2.66 to 0.05)	0.0585
Change at 6 months	n=3035	n=1562	n=1473		
Physical limitation	2.3±0.4 (1814)	1.6±0.6 (937)	3.0±0.6 (877)	−1.74 (−3.34 to −0.14)	0.0329
Angina stability	13.0±0.6 (2833)	13.4±0.9 (1462)	12.5±0.9 (1371)	1.27 (−0.27 to 2.80)	0.1059
Angina frequency	18.7±0.4 (2895)	18.3±0.6 (1498)	19.2±0.6 (1397)	−1.55 (−2.85 to −0.25)	0.0198
Treatment satisfaction	−4.7±0.3 (2872)	−5.0±0.4 (1485)	−4.3±0.4 (1387)	−0.97 (−2.14 to 0.21)	0.1060
Quality of life	17.0±0.4 (2865)	15.5±0.6 (1484)	18.6±0.6 (1381)	−3.48 (−4.95 to −2.01)	<0.0001

Mean±SE (n).

Comparisons of the symptomatic improvement between the two trial groups demonstrated few early (6 weeks) differences, but by 6 months, improvements in physical limitation, angina frequency and especially quality of life were less marked in those assigned to CTCA (table 1). These differences did not reflect bias in non-responders (see online supplementary table 2), were generally small in magnitude (<4%) but were also consistently seen in the Short Form 12 responses (table 2). Responder analysis suggested that CTCA was associated with more patients experiencing an improvement in the stability of their symptoms at 6 weeks and frequency of their symptoms at 6 months (see online supplementary table 3).

**Table 2:** Medical outcomes study Short Form 12 (SF-12)

	Standard care+CTCA	Standard care	Difference (95% CIs)	p Value (for difference)
SF-12 physical summary				
Baseline	44.2±0.2 (1838)	44.0±0.2 (1829)	0.1 (−0.5 to 0.8)	0.70
6 weeks	44.3±0.3 (1649)	44.5±0.3 (1562)	−0.2 (−0.9 to 0.6)	0.66
6 months	45.0±0.3 (1566)	46.0±0.3 (1478)	−1.0 (−1.8 to −0.2)	0.01
SF-12 mental summary				
Baseline	46.1±0.3 (1838)	46.7±0.3 (1829)	−0.6 (−1 3 to 0.2)	0.12
6 weeks	47.2±0.3 (1649)	47.0±0.3 (1562)	0.2 (−0.6 to 1 0)	0.57
6 months	47.8±0.3 (1566)	48.6±0.3 (1478)	−0.8 (−1 6 to −0.0)	0.05
SF-6D utility index				
Baseline	0.70±0.003 (1882)	0.71±0.003 (1871)	−0.01 (−0.01 to 0.00)	0.24
6 weeks	0.72±0.003 (1678)	0.72±0.004 (1591)	0.00 (−0.01 to 0.01)	0.94
6 months	0.73±0.004 (1596)	0.74±0.004 (1505)	−0.01 (−0.02 to 0.00)	0.05

Mean±SE (n).

SF-12 physical summary, SF-12 mental summary and SF-6D utility index are subsections of the SF-12 questionnaire.

CTCA, CT coronary angiography.

### Influence of the change in diagnosis

Between the initial clinic consultation and 6 weeks, the diagnosis of coronary heart disease was changed in 28% of patients who underwent CTCA compared with 1% of those who received standard care alone (p<0.001), and the diagnosis of angina due to coronary heart disease changed in 23% and 1% (p<0.001), respectively. The change in the diagnosis of coronary heart disease was associated with differing responses to the changes in symptoms (table 3). In general, improvements in symptoms of physical limitation, angina frequency and quality of life were reduced when a new diagnosis of angina pectoris due to coronary heart disease was made. Similar findings were observed for changes to the diagnosis of coronary heart disease (table 4).

**Table 3:** Changes in Seattle Angina Questionnaire after 6 weeks and 6 months according to how the diagnosis of angina pectoris due to coronary heart disease changed in patients allocated to CT coronary angiography

	Diagnosis of angina pectoris due to CHD refuted	New diagnosis of angina pectoris due to CHD	Unchanged diagnosis	p Value*
Total patients (n)	310	171	1588	
Change at 6 weeks (n)	277	158	1320	
Physical limitation	−2.4±1.3 (166)	−3.3±1.3 (100)	0.21±0.6 (816)	0.0053
Angina stability	20.4±2.0 (261)	8.9±2.9 (146)	16.8±1.0 (1230)	0.4434
Angina frequency	7.9±1.6 (266)	7.2±1.8 (152)	12.4±0.6 (1266)	<0.0001
Treatment satisfaction	−7.8±1.0 (264)	−7.9±1.4 (150)	−6.7±0.5 (1261)	0.4938
Quality of life	9.7±1.5 (261)	4.1±1.6 (151)	9.0±0.6 (1269)	0.0137
Change at 6 months (n)	252	139	1171	
Physical limitation	1.9±1.6 (158)	−3.5±2.0 (84)	2.1±0.7 (695)	0.0121
Angina stability	18.1±2.0 (242)	7.8±3.3 (128)	13.0±1.1 (1092)	0.8572
Angina frequency	15.8±1.6 (245)	13.4±2.2 (134)	19.5±0.7 (1119)	<0.0001
Treatment satisfaction	−6.8±1.3 (238)	−7.3±1.7 (131)	−4.4±0.5 (1116)	0.0510
Quality of life	15.8±1.6 (237)	10.4±2.0 (132)	16.0±0.7 (1115)	0.0060

Mean±SE (n).

Changes in diagnosis reflect those recorded by the attending clinician at 6 weeks postclinic attendance.

*Analysis of variance across the three groups.

CHD, coronary heart disease.

**Table 4:** Changes in Seattle Angina Questionnaire after 6 weeks and 6 months according to how the diagnosis of coronary heart disease changed in patients allocated to CT coronary angiography

	Diagnosis of CHD refuted	New diagnosis of CHD	Unchanged diagnosis	p Value
Total patients (n)	423	166	1480	
Change at 6 weeks (n)	376	155	1224	
Physical limitation	0.3±1.2 (226)	−2.0±1.8 (101)	−0.6±0.6 (755)	0.8198
Angina stability	17.8±1.7 (345)	7.0±3.1 (147)	17.6±1.1 (1145)	0.1171
Angina frequency	11.3±1.4 (359)	8.1±1.7 (151)	11.6±0.7 (1174)	0.4520
Treatment satisfaction	−6.6±0.9 (355)	−8.6±1.4 (148)	−6.9±0.5 (1172)	0.6693
Quality of life	11.3±1.2 (354)	4.5±1.8 (151)	8.4±0.6 (1176)	0.0819
Change at 6 months (n)	341	137	1084	
Physical limitation	3.1±1.4 (206)	−3.2±2.0 (81)	1.7±0.7 (650)	0.1036
Angina stability	16.3±1.8 (318)	6.4±3.5 (125)	13.4±1.1 (1019)	0.6374
Angina frequency	18.6±1.5 (330)	10.3±2.1 (129)	19.3±0.7 (1039)	0.0006
Treatment satisfaction	−5.0±1.1 (321)	−8.3±1.5 (127)	−4.6±0.5 (1037)	0.1576
Quality of life	18.0±1.3 (321)	8.6±2.1 (129)	15.6±0.7 (1034)	0.0164

Mean±SE (n).

Changes in diagnosis reflect those recorded by the attending clinician at 6 weeks postclinic attendance.

CHD, coronary heart disease.

### Determinants of symptomatic change

In univariable analyses adjusted for baseline scores (table 5), the main predictor of symptomatic improvement in patients undergoing CTCA was the baseline score: those with the lowest score made the most improvement. However, there were other determinants that predicted improvements in symptoms. For both physical limitation and angina stability, moderate (50–70% stenosis) non-obstructive disease was the least favourable and was associated with deteriorating (physical limitation, −4±1.3) or lower gains (angina stability, 10±1.4) than patients with normal (6±1.0 and 15±1.0, respectively), mild non-obstructive (1±1.2 and 15±1.3) or obstructive coronary artery disease (2±1.7 and 16±1.7 for single-vessel disease).

**Table 5:** Univariable mixed models to investigate how symptom-related variables could help explain the changes in Seattle Angina Questionnaire scores at 6 months

	Physical limitation	Angina stability	Angina frequency	Treatment satisfaction	Quality of life
Baseline score	p<0.0001	p<0.0001	p<0.0001	p<0.0001	p<0.0001
Age (years)	p=0.0131	p=0.0743	p=0.3676	p=0.0006	p=0.6478
18–59	2.4±0.8	14.5±0.8	–	−6.1±0.6	–
60–75	0.7±0.9	12.3±0.8	–	−3.9±0.6	–
Gender	p=0.0839	p=0.0546	p=0.5861	p=0.2014	p=0.1543
Female	0.4±0.9	14.7±0.9	–	–	16.4±0.8
Male	2.4±0.7	12.5±0.7	–	–	14.8±0.7
History of coronary heart disease	p=0.0204	p=0.0142	p=0.1495	p=0.6387	p=0.0010
Yes	−2.3±1.8	9.3±1.8	16.3±1.5	–	10.2±1.7
No	2.1±0.6	13.9±0.6	18.6±0.5	–	16.1±0.6
Exercise ECG	p=0.0410	p=0.6896	p=0.0007	p=0.1514	p=0.0104
Normal	2.1±0.8	–	19.9±0.6	−4.2±0.6	16.7±0.7
Inconclusive	−0.7±1.6	–	15.1±1.3	−5.5±1.2	12.9±1.5
Abnormal	4.5±1.5	–	15.6±1.3	−5.6±1.1	15.4±1.4
Not performed	−0.9±1.6	–	17.2±1.2	−6.9±1.1	11.9±1.5
CTCA-defined coronary heart disease	p<0.0001	p=0.0167	p<0.0001	p=0.0907	p<0.0001
Normal (<10%)	5.9±1.0	14.6±1.0	21.8±0.8	−3.9±0.8	20.1±0.9
Mild non-obstructive (10–50%)	0.9±1.2	15.4±1.3	18.3±1.1	−4.5±.09	15.0±1.2
Moderate non-obstructive (50–70%)	−4.3±1.3	9.5±1.4	14.1±1.2	−7.5±1.0	10.7±1.3
Single-vessel disease	1.8±1.7	16.1±1.7	16.4±1.4	−4.7±1.3	14.5±1.6
Two-vessel disease	0.7±2.2	13.4±2.1	17.4±1.8	−2.9±1.6	14.0±2.0
Three-vessel disease	1.4±2.1	12.4±2.2	15.8±1.8	−5.5±1.6	11.2±2.0
Coronary revascularisation*	p=0.1164	p=0.1316	p=0.4093	p=0.0437	p=0.8591
Yes	4.3±1.8	15.8±1.7	–	−2.5±1.3	–
No	1.3±0.6	13.1±0.6	–	−5.3±0.4	–
Preventative therapy	p=0.0066	p=0.8253	p=0.1100	p=0.0383	p<0.0001
Cancelled	7.1±2.9	–	23.0±2.4	−1.6±2.2	25.9±2.7
New therapy initiated	−2.0±1.5	–	15.7±1.2	−7.14±1.1	11.7±1.4
No change	2.0±0.6	–	18.6±0.5	−4.8±0.5	15.7±0.6

Mean±SE.

All analyses were adjusted for the baseline score.

p Values refer to subcategories that predicted changes in the Seattle Angina Questionnaire at 6 months. The magnitude of changes across individual variables are given where p≤0.15.

*Within 6 months of randomisation.

CTCA, CT coronary angiography.

In multivariate analyses, improvements in angina frequency were predicted by the change in diagnosis of coronary heart disease (p=0.0498). Where a positive baseline diagnosis of angina due to coronary heart disease was refuted by the CTCA, most improvements were seen in those where CTCA demonstrated normal (22±2.3, p<0.001) or mild (22±3.9, p<0.001) coronary artery disease. In contrast, where a baseline diagnosis of angina due to coronary heart disease was not thought to be present but was subsequently corrected to confirm its presence by the CTCA, symptoms improved most if patients were found to have obstructive two-vessel or three-vessel disease (18±5.2 and 20±7.3, respectively).

Treatment satisfaction was excellent at baseline and fell slightly across all groups. However, it fell less in those aged 60–75 years (−4±0.6) than those aged 18–59 years (−6±0.6). Other predictors of a lower decline in treatment satisfaction included a normal exercise ECG (−4±0.6 vs −6±1.1), undergoing coronary revascularisation (−3±1.3 vs −5±0.4) and cancellation of preventative therapies (−2±2.2 vs −5±0.5 (no change) and −7±1.1 (new therapy initiated)).

Improvements in quality of life were predicted by a number of factors. Predictors of reduced improvements in quality of life included history of coronary heart disease (10±1.7 vs 16±0.6), post-CTCA diagnosis of coronary heart disease (three-vessel coronary heart disease (11±2.0) and moderate non-obstructive coronary heart disease (11±1.3) compared with normal (20±0.9) or single-vessel coronary heart disease (15±1.6)) and exercise ECG (abnormal (15±1.4), inconclusive (13±1.5) or not performed (12±1.5) compared with normal (17±0.7)). Interestingly, cancellation of preventative therapy was associated with the most improvement in quality of life (26±2.7) compared with no change (16±0.6) or initiation of new therapy (12±1.4).

## Discussion

In patients with suspected angina due to coronary heart disease, attendance at the cardiology outpatient clinic is associated with high levels of immediate treatment satisfaction and symptomatic improvement. While markedly clarifying the diagnosis for the attending clinician, CTCA was associated with a small attenuation of improvements in overall symptoms and quality of life compared with standard care alone. Poorer symptom outcomes appeared to be attributable to changes in the diagnosis and especially the detection of moderate non-obstructive coronary artery disease. However, the greatest improvements in symptoms were seen in those with normal exercise tolerance, normal coronary arteries and those who had preventative therapies cancelled.

Previous head-to-head comparisons of CTCA with exercise stress testing have demonstrated either neutral findings or improved symptoms and quality of life immediately after investigation.^8^ ^9^ Indeed, many studies have suggested a high degree of short-term patient satisfaction and preference for CTCA.^10–13^ Here, we have investigated the additional effect of CTCA on the improvements in patients' symptoms following attendance at the rapid access chest pain clinic that included an exercise test in the majority (85%) of attendances. We have made a number of notable and novel observations, some of which may initially seem counterintuitive. How can CTCA attenuate the 6-month improvement in symptoms when it clarifies the diagnosis, enhances the appropriate use of invasive coronary angiography, alters preventative and anti-anginal treatments, and reduces the incidence of myocardial infarction?^1^ ^2^ ^14^

It should be remembered that two-thirds of patients who attended the cardiology clinic with chest pain were not diagnosed with angina pectoris due to coronary heart disease. Moreover, CTCA increased the diagnosis of coronary heart disease but decreased the diagnosis of angina due to coronary heart disease. Patients diagnosed with non-cardiac chest pain have higher levels of anxiety than those diagnosed with cardiac pain since their symptoms are perceived to be less controllable or understandable.^15^ For these patients, the outcome of a CTCA will commonly include a diagnosis of covert non-obstructive coronary heart disease with the consequence that patients may become more confused and concerned about their future health and well-being. This would also explain the association of reduced improvements in quality of life with the new prescription of preventative therapies. It is therefore perhaps not unsurprising that CTCA was associated with worse symptomatic outcomes for such patients. Intriguingly, patients with moderate non-obstructive coronary artery disease had the worst symptomatic outcomes across all five domains of the Seattle Angina Questionnaire, even in comparison to patients with obstructive triple-vessel coronary heart disease. Ultimately for these patients, CTCA did not provide the reassurance they were looking for nor did it identify the cause of their presenting symptoms of chest pain.

For some patients, CTCA was associated with better symptomatic outcomes. This predominantly related to patients who had an initial clinic diagnosis of angina pectoris due to coronary heart disease where the CTCA was able to demonstrate the absence of coronary heart disease. This led to the cancellation of unnecessary anti-anginal and preventative therapies, and was associated with the better improvements in symptoms and quality of life. In addition, it is important to recognise that despite high levels of treatment satisfaction the majority of patients who are diagnosed with non-cardiac chest pain continue to be concerned that there is an underlying cardiac cause of their chest pain.^3^ The documentation of normal coronary arteries by CTCA was associated with the greatest improvements in symptoms and quality of life, suggesting that CTCA may be particularly valuable to provide reassurance for patients who remain anxious about the possibility of covert coronary heart disease. Of course, should the CTCA demonstrate non-obstructive coronary heart disease, this will only serve to increase their anxiety.

Which patients should we select for CTCA to improve symptoms? We explored the determinants of symptomatic improvements in patients undergoing CTCA. Perhaps unsurprisingly and consistent with previous studies,^16^ those with the worst symptoms derived most benefit from clinic attendance and investigation. Beyond this, important predictors included the absence of a history of coronary heart disease, normal exercise tolerance testing, normal coronary arteries on CTCA, undergoing coronary revascularisation and cancellation of preventative therapies. This would therefore suggest that the major symptomatic benefit of CTCA is in the demonstration of normal coronary arteries in those who are being treated with inappropriate preventative therapies, have good exercise tolerance and do not have known coronary heart disease. This is in keeping with the current National Institute for Health and Care Excellence guidelines on the management of chest pain of recent onset.^17^

Much like the diagnosis of cancer, the new diagnosis of coronary heart disease, a potentially life-threatening condition, is unlikely to improve quality of life.^18^ The increased diagnosis of non-obstructive coronary heart disease by CTCA is the main driver for the attenuation of symptomatic improvement in patients presenting to the cardiology clinic. This is however counterbalanced by the enhanced improvement in symptoms for those patients with normal coronary arteries who are taking unnecessary preventative therapies, as well as the more appropriate use of invasive coronary angiography, secondary prevention and coronary revascularisation in those with unrecognised obstructive coronary heart disease.^2^ Ultimately, it is important to balance the small increase in anxiety caused by the identification of covert coronary artery disease, and the major benefits of initiating appropriate symptomatic and preventative therapy to avoid future cardiac events given the reduction in overall rates of subsequent myocardial infarction.^1^ ^2^ ^14^

We acknowledge that the magnitude of the differences between the study groups are small and often less than five points of a 100-point scale. However, these are population differences and such small changes can be important. For example, while a 2–4 mm Hg fall in blood pressure is very small on an individual level, this is associated with 28% reduction in the rate of stroke and 22% reduction in death at a population level.^19^ Furthermore, consistent with the heterogeneous profile of the study population, we found more marked differences within different groups especially with respect to the extent of coronary artery disease. This is perhaps not unsurprising given that CTCA defines this aspect of the patient's profile and increased the diagnosis of coronary heart disease.

We should acknowledge some limitations of our study. We do not have information on the psychological status of our patients nor do we have detailed information on coronary microvascular function. Some of the participants may have had angina due to coronary microvascular disease, and this can affect 10–30% of patients with angina and non-obstructive coronary heart disease^20^ ^21^ and cessation of angina therapy may have led to a deterioration in symptoms. We also cannot discount misclassification of disease. CTCA provides a non-invasive imaging assessment of the anatomical severity of coronary artery disease and is less accurate than invasive angiography with quantitative analysis or functional techniques, such as fractional flow reserve. However, it is reassuring that in patients who underwent CTCA the frequency of normal coronary angiography was reduced by two-thirds and obstructive disease was substantially more common at the time of invasive angiography.^2^

In conclusion, CTCA attenuates the overall improvement in symptoms of patients presenting with suspected angina pectoris due to the increased diagnosis of coronary heart disease. CTCA is associated with better symptomatic outcomes in patients proven to have normal coronary arteries who had preventative therapies cancelled, while poorer symptom outcomes were seen for those with undiagnosed non-obstructive coronary heart disease for whom preventative therapies were initiated. These effects on symptoms need to be balanced against the potential benefits of improved focused clinical management and reductions in the rates of myocardial infarction.^1^ ^2^ ^14^

**Key messages**

**What is already known on this subject?**

- Previous head-to-head comparisons of CT coronary angiography (CTCA) with exercise stress testing have demonstrated either neutral findings or improved symptoms and quality of life immediately after investigation. Indeed, many studies have suggested a high degree of short-term patient satisfaction and preference for CTCA.

**What might this study add?**

- CTCA is associated with a small attenuation of the improvements in overall symptoms and quality of life compared with standard care alone. This appears to be attributable to changes in the diagnosis and especially the detection of moderate non-obstructive coronary artery disease. The greatest improvements in symptoms are seen in those with normal exercise tolerance, normal coronary arteries and those who have preventative therapies cancelled.

**How might this impact on clinical practice?**

- CTCA improves symptoms by identifying patients with normal or obstructive coronary heart disease. In untreated patients with atypical symptoms, clinicians need to discuss the implications of potential CTCA findings including non-obstructive disease that would mandate preventative therapies to avoid future coronary heart disease events.
