# Supplementary material for: Symptoms and quality of life in patients with suspected angina undergoing CT coronary angiography: a randomised controlled trial
Source: Heart. 2017 Feb 28;103(13):995–1001. doi: 10.1136/heartjnl-2016-310129 (PMC5529983; doi:10.1136/heartjnl-2016-310129)
Supplement: supplementary tables [file heartjnl-2016-310129supp001.pdf]

Supplementary Table 1

Seattle Angina Questionnaire scores at each time point.

	Standard Care + CTCA	Standard Care	Difference (95% Confidence Intervals)	P value (for difference)
**Physical Limitation**				
**Baseline**	73·37±0·56 (1618)	73·96±0·56 (1588)	-1·19 (-2·80 to 0·41)	0·144
**Six Weeks**	73·35±0·70 (1266)	74·10±0·74 (1190)	-0·75 (-2·74 to 1·24)	0·461
**Six Months**	76·14±0·71 (1123)	78·71 ±0·56 (1053)	-2·56 (-4·59 to -0·54)	0·013
**Angina Stability**				
**Baseline**	44·44±0·63 (1957)	44·32±0·64 (1936)	0·12 (-1·63 to 1·88)	0·889
**Six Weeks**	67·48±0·57 (1721)	60·54 ±0·59 (1642)	0·94 (-0·67 to 2·55)	0·252
**Six Months**	58·26±0·54 (1537)	56·92 ±·53 (1448)	1·34 (-0·14 to 2·82)	0·077
**Angina Frequency**				
**Baseline**	67·67±0·49 (2007)	67·76±0·50 (1968)	-0·09 (-1·47 to 1·29)	0·897
**Six Weeks**	79·28±0·55 (1727)	80·05±0·57 (1643)	-0·77 (-2·31 to 0·78)	0·330
**Six Months**	86·27±0·49 (1538)	87·76±0·50 (1451)	-1·49 (-2·86 to -0·12)	0·033
**Treatment Satisfaction**				
**Baseline**	92·21±0·27 (1999)	92·30±0·29 (1964)	-0·09 (-0·86 to 0·68)	0·817
**Six Weeks**	85·59±0·42 (1727)	85·38±0·46 (1638)	0·21 (-1·00 to 1·42)	0·733
**Six Months**	87·68±0·44 (1529)	88·79±0·45 (1440)	-1·11 (-2·35 to 0·13)	0·079
**Quality of Life**				
**Baseline**	54·77±0·47 (2009)	54·60±0·49 (1972)	0·18 (-1·15 to 1·51)	0·795
**Six Weeks**	63·99±0·57 (1723)	65·19±0·59 (1641)	-1·19 (-2·80 to 0·41)	0·144
**Six Months**	71·09±0·58 (1524)	74·54±0·58 (1434)	-3·45 (-5·06 to -1·84)	<0·001

Mean±standard error of the mean (n)

Supplementary Table 2

Baseline Seattle Angina Questionnaire scores for patients who did not return respond to the questionnaires at 6 weeks and 6 months.

	**Standard Care + CTCA**	**Standard Care**	**Difference** (95% Confidence Intervals)	**P value** (for difference)
**Not Responding at Six Weeks**				
**Physical Limitation**	73·25±0·97 (536)	73·78±0·92 (594)	-0·52 (-3·14 to 2·09)	0·695
**Angina Stability**	42·97± 1·57 (320)	43·60± 1·51 (383)	-0·63 (-4·92 to 3·65)	0·771
**Angina Frequency**	66·69± 1·31 (323)	65·93± 1·18 (388)	0·76 (-2·69 to 4·21)	0·666
**Treatment Satisfaction**	90·26±0·74 (324)	90·93±0·72 (392)	-0·67 (-2·71 to 1·36)	0·516
**Quality of Life**	52·44± 1·24 (328)	51·19± 1·12 (392)	1·25 (-2·03 to 4·53)	0·455
**Not Responding at Six Months**				
**Physical Limitation**	72·31±0·90 (681)	72·76±0·86 (711)	-0·45 (-2·89 to 1·98)	0·715
**Angina Stability**	43·03± 1·25 (495)	43·94± 1·21 (565)	-0·91 (-4·34 to 2·52)	0·604
**Angina Frequency**	67·49±0·96 (509)	65·45±0·98 (571)	2·04 (-0·67 to 4·74)	0·140
**Treatment Satisfaction**	90·71±0·57 (514)	90·04±0·62 (577)	0·68 (-0·98 to 2·34)	0·425
**Quality of Life**	53·00±0·94 (525)	51·33±0·92 (591)	1·67 (-0·91 to 4·25)	0·205

Mean±standard error of the mean (n)

Supplementary Table 3

Responder analysis at 6 weeks and 6 months according to treatment allocation. A responder was defined as an increase in the Seattle Angina Questionnaire score of ≥10.[5]

	Standard Care + CTCA	Standard Care	Odds Ratio (95% Confidence Intervals)	P value
**Six Weeks**				
**Physical Limitation**	192 (9·3%)	196 (9·5%)	0·982 (0·796-1·212)	0·868
**Angina Stability**	816 (39·4%)	756 (36·5%)	1·140 (1·004-1·294)	0·043
**Angina Frequency**	1036 (50·0%)	979 (47·2%)	1·120 (0·991-1·266)	0·071
**Treatment Satisfaction**	152 (7·3%)	151 (7·3%)	1·005 (0·795-1·271)	0·966
**Quality of Life**	699 (33·7%)	689 (33·2%)	1·026 (0·901-1·169)	0·695
**Six Months**				
**Physical Limitation**	255 (12·3%)	233 (11·2%)	1·117 (0·924-1·351)	0·254
**Angina Stability**	664 (32·0%)	617 (29·8%)	1·124 (0·984-1·284)	0·086
**Angina Frequency**	1118 (53·9%)	1045 (50·4%)	1·155 (1·021-1·308)	0·022
**Treatment Satisfaction**	185 (8·9%)	178 (8·6%)	1·037 (0·835-1·287)	0·745
**Quality of Life**	794 (38·3%)	819 (39·5%)	0·950 (0·838-1·078)	0·431

N (%)

Analysis was adjusted for minimisation variables and centre allocation.
